# Editorial: Molecular and cellular mechanisms of sensory functions in insect models, volume II

**DOI:** 10.3389/fnmol.2024.1443041

**Published:** 2024-06-17

**Authors:** Takaaki Sokabe, Jia Huang, Youngseok Lee

**Affiliations:** ^1^Thermal Biology Group, Exploratory Research Center on Life and Living Systems (ExCELLS)/Section of Sensory Physiology, Center for Genetic Analysis of Behavior, National Institute for Physiological Science, National Institutes of Natural Sciences, Okazaki, Japan; ^2^Institute of Insect Sciences, Zhejiang University, Hangzhou, China; ^3^Department of Bio & Fermentation Convergence Technology, Kookmin University, Seoul, Republic of Korea

**Keywords:** sensory function, *Drosophila melanogaster*, receptor, ion channel, signaling molecule, molecular function, neuron, behavior

Sensory systems are indispensable for all organisms to receive dynamic information from their surrounding environment. A diverse set of sensory and signaling molecules has been identified; however, it remains largely uncharacterized how they act, maintain the functions, and interact with each other. Fruit flies (*Drosophila melanogaster*) are a versatile model for understandings the bases of sensory functions due to their simple yet robust behaviors that rely on their simple neural circuits. Therefore, these small insects have been used over a century for basic neuroscience research and many other research fields as well. In this Research Topic volume II regarding sensory mechanisms, a variety of articles were provided by dedicated contributors, including the diversity and complexity of sensory functions with useful new methodologies in *Drosophila*.

Mariette et al. developed a new measurement tool for the functional study of odorant receptors (ORs) named Transcuticular Calcium Imaging (TCI). The authors adopted an empty neuron system, in which endogenous ORs have been removed in specific sensilla to express exogenous ORs for tests. TCI just requires a conventional fluorescence microscope equipped with a camera to capture GCaMP-derived calcium signals through intact cuticular of antennae. They demonstrated that the specificity and sensitivity of calcium responses of two ORs from cotton leafworm were sufficiently high and nearly identical to those obtained by a single sensillum recording. Compared to the labor-intensive electrophysiological recording method that holds only a single fly, TCI is equivalently sensitive, and advantageous in terms of recording duration reaching hours and the simultaneous testing of multiple insects, thus serving as an alternative handy tool for OR deorphanization.

Chemical treatments such as insecticides and repellents have been major strategies against insect pests. However, due to a limited understanding of the mechanisms behind insect avoidance behaviors, the development of insect repellents has been delayed. Sato et al. found that 2-methylthiazoline (2MT), a Transient Receptor Potential (TRP) A1 stimulant, acts as a potent insect repellent in *Drosophila*. 2MT induces aversive behaviors through olfactory, gustatory, and nociceptive sensory pathways. It targets TRPA1 via specific cysteine residues conserved across many insect pest species. This research could advance the development of novel insect repellents by highlighting TRP channels and other receptor types as promising targets.

Environmental temperature is a universal physical parameter that affects survival and other life history of organisms. Evans et al. utilized larvae as a model for systematic analysis of the cellular and behavioral responses to temperature across development. They measured multiple parameters of locomotion and showed development-dependent increase in run speed and decrease in turn rate. On a thermal gradient condition, older larvae displayed quicker navigation toward the warmer side. However, the efficiency of the navigation decisions stayed the same across development, suggesting that thermotaxis depends on the combination of the decision-based rules and physical capabilities. They also demonstrated that larvae at all stages and their cooling sensors were able to respond to extremely small temperature changes, as small as 0.001°C/s cooling or 0.01°C absolute change. This study provides a basic set of behavioral parameters for future studies concerning animals' thermotaxis.

Cilia are thin protrusions from the cell body and play diverse functions from bacteria to ciliated cells such as sperm and sensory neurons. This unique structure is formed and maintained by reciprocal trafficking of components along the axoneme, which is called intraflagellar transport (IFT). Sharma et al. identified an IFT-associated cytoplasmic dynein heavy chain (Dync2h1, a.k.a. Dhc1b) encoded by *beethoven* (*btv*) as a responsible gene for retrograde transport in *Drosophila*. The *btv* mutant displayed severe and age-dependent impairments in auditory response in adult antennae, along with defects in ciliary biogenesis and degeneration in chordotonal organs. Also, the electrophysiological property of the external sensory neurons with a short cilium were altered by *btv* mutation. As an IFT component, *btv* appeared to contribute to clearing of IFT complex proteins NompB in sensory cilia of chordotonal organ and RempA in external sensory organ dendrites. This evidence proves that protein recycling in cilia is crucial for the morphology and mechanical responsiveness of the ciliated sensory neurons.

Color vision enhances the identification of food sources, conspecifics, predators, and prey, supporting a wide range of behaviors. Evolutionarily distant animals, such as monkeys and flies, may utilize similar circuit mechanisms for the initial stage of color opponent processing. In *Drosophila*, Dm9 neurons have been proposed as functional homologs of horizontal cells in the vertebrate outer retina. Schnaitmann et al. highlight a crucial role for Dm9 neurons in color opponent processing and suggest an additional role in regulating light adaptation in inner photoreceptors. These findings on Dm9 neurons parallel the versatile functions of horizontal cells in the vertebrate retina, underscoring their importance in visual processing across species.

Aminergic nuclei are clusters of neurons that primarily use monoamine neurotransmitters for communication. These clusters are generally composed of relatively small numbers of cells with extensive projection patterns in mammals, and the potential heterogeneities among aminergic neurons remain poorly understood. Rohrbach et al. dissected the functional diversity of aminergic neurons within a specific neuroanatomic cluster. They mapped the projections of individual aminergic neurons that innervate the female reproductive tract and identified potential differences in their function and excitability. This research establishes a framework for studying the roles of different aminergic neurons within an anatomically defined cluster and how each may contribute to the overall function of the cluster.

Sexual dimorphism in behavior is primarily a result of distinct neural circuit organization between males and females. The genes *fruitless* (*fru*) and *doublesex* (*dsx*) play crucial roles in shaping these sexually dimorphic neural circuits. Sato and Yamamoto focused on three neural groups, namely the P1 cluster, aSP-f and aSP-g cluster pairs, and aDN cluster, to elucidate the causal relationships between behavior and neural characteristics influenced by *fru* and/or *dsx* expression. The aSP-f, aSP-g, and aDN clusters exemplify instances where *fru* or *dsx* autonomously regulate neurite structures, leading to distinct male and female neural configurations. They proposed that the *fru* and *dsx* gene products act as terminal selectors in shaping sexually dimorphic neuronal wiring, maintaining sex-typical chromatin states. These genes coordinate the transcription of effector genes, impacting the structures of individual neurons, and overseeing processes related to the survival and death of cells.

These articles demonstrate the usefulness of *Drosophila* as a model for studying multiple aspects of sensory functions by providing valuable insights into the fundamental mechanisms of sensory processes and related behaviors. These findings should benefit studies in higher organisms including humans.

## Author contributions

TS: Writing – original draft, Writing – review & editing. JH: Writing – review & editing. YL: Writing – review & editing.

